# Gender differential impact of bereavement on health outcomes: evidence from the China Health and Retirement Longitudinal Study, 2011–2015

**DOI:** 10.1186/s12888-020-02916-2

**Published:** 2020-10-22

**Authors:** Zhuo Chen, Jiahui Ying, Justin Ingles, Donglan Zhang, Janani Rajbhandari-Thapa, Ruoxi Wang, Kerstin Gerst Emerson, Zhanchun Feng

**Affiliations:** 1 College of Public Health University of Georgia, Wright Hall 305B, 100 Foster Rd, Athens, GA 30602 USA; 2 University of Nottingham Ningbo China, Ningbo, 315100 Zhejiang China; 3 Huazhong University of Science and Technology of China, Wuhan, 430074 Hubei China

**Keywords:** Bereavement, Center for Epidemiologic Studies Depression Scale, Self-assessed health, Survivor benefits, China

## Abstract

**Background:**

Bereavement is the experience of an individual following the death of a person of significance to the individual, most often referring to the spouse. Increased morbidity, health care utilization, and mortality are known to be associated with bereavement. Given China’s growing population of older adults, there is a critical need to assess the health consequences of bereavement.

**Method:**

We use data from the China Health and Retirement Longitudinal Study to examine the impact of bereavement on mental health and quality of life among a sample of mid- and older-aged adults. We use propensity score matching to construct a matching sample and difference-in-differences method to estimate the impact of bereavement on mental health and self-assessed health.

**Results:**

We find bereavement is associated with increased depression symptoms among women (1.542 point or 0.229 standard deviations of Center for Epidemiologic Studies Depression (CES-D) 10 score) but not consistently for men over time. No statistically significant effect of bereavement on self-assessed health is found.

**Conclusions:**

Our results show a harmful impact of bereavement on mental health among older women in China and point to the need for a comprehensive policy on survivor benefits in China, particularly for rural older women.

## Background

Family protects members from the uncertainties of life. Bereavement, the death of a family member, can be a significant life event that leaves survivors with a traumatic grief experience [1, 2]. Bereavement can involve the loss of a parent, spouse, sibling, or child. One’s risk of exposure to spousal death increases with age. Evaluating the consequence of a late-life spousal bereavement has important implications for research and policymaking, particularly for countries with an aging population. In China, the proportion of older adults is projected to increase to 26% by 2050, exceeding that of most European countries [3]. Examining the impact of spousal bereavement in the old age in China is a critical and timely topic.

### Bereavement and health outcomes

The health consequences of spousal bereavement can be classified into three categories: mortality, morbidity, and mental health. Empirical literature shows evidence of an association between spousal bereavement and increased mortality rate, especially for the first 6 months after spousal bereavement [4, 5]. Examining data from Wales, UK, Rees and Lutkins find that among all close relatives, the greatest increase of mortality is found among widows and widowers: the mortality rate of the survived spouse was more than 10 times greater than that of the matched controls [6]. With an analysis of 9 years of longitudinal follow-up data in England and Wales of 4486 widowers of 55 years of age and older, Parkes and colleagues report a mortality rate for widowers that was 40% above the expected rate for married men of the same age [7]. The greatest increase in mortality was found during the first 6 months in the widowers dying from heart disease. Berkman and Syme have analyzed the 1965 Human Population Laboratory survey of a random sample of 6928 adults in Alameda County, California. They conclude that people who lacked social and community ties were more likely to die in the follow-up period than those with more extensive contacts [8]. The relationship is independent of self-reported physical health status, year of spousal death, socioeconomic status, and health practices.

Beyond mortality, investigating the effect of late-life spousal bereavement on the morbidity among the bereaved survivors is also essential. Cardiovascular disease is shown to be a potential risk for individuals in early bereavement [9]. Other physical health risks associated with bereavement include weight loss, decreased nutritional intake, immune system impairment, and increased illness rates [10, 11]. Bereavement may also lead to risky behaviors. For instance, bereaved men are found to endure higher risks of alcohol-related problems [12].

Bereavement is linked with mental health risks [11], including complicated grief and bereavement-related depression [13]. King and colleagues use a UK primary care database to investigate the bereavement effect for the ones whose cohabitee died from cancers. They find the bereaved patients were more likely to receive a prescription for an antidepressant or hypnotic medication and to attend their general practices both before and after the death of the cohabitee [14]. Among a sample of Chinese American older adults, the loss of a close relative or friend was associated with more stress, especially among women. In contrast, the loss of children or grandchildren was linked to stronger anxiety [15]. The existing literature studying the association between bereavement and mental health outcomes among Chinese populations have examined bereavements associated with the loss of an only-child [16, 17] or the sudden death of a family member(s) in natural disasters [17–20]. Among survivors of a disastrous earthquake in 1999 in middle Taiwan, bereaved survivors had lower scores for optimism, but higher scores for depressed mood, loneliness, and anxiety than those who had no loss of family members [19].

### Causal pathways and gender difference

Multiple causal pathways exist for bereavement to affect mental and physical health. First, grief is associated with increased risks of mental health issues [2], which could lead to physical health problems [14]. Second, loneliness after bereavement is associated with an increased likelihood of depression and other mental health issues [21, 22]. Third, among surviving spouses without employment or pension, bereavement may lead to reduced income, which affects nutrition, housing, and healthcare decisions. However, bereavement may improve health for surviving spouses who have served as a caretaker providing the strenuous end-of-life care for the deceased.

Gender difference in the impact of bereavement on health outcomes exists. Stahl et al. have analyzed the Cardiovascular Health Study data in the U.S. and find a significant interaction of gender with widowhood [23]. Their results suggested that bereavement decreased the risk of mortality in women but increased the risk of mortality in men. Analyzing 518,240 couples who were enrolled in Medicare in 1993, Christakis and Allison have reported the hospitalization of a spouse was associated with an increased risk of death. The increase was the highest for men when the spouse was hospitalized for stroke, congestive heart failure, hip fracture, psychiatric disease, or dementia, but the effect on women was not significant [24]. Ho and colleagues have used a clinical measure to assess the grief reactions to the loss of a family member among 180 bereaved residents in Hong Kong [25]. They find that women had stronger grief reactions than men. In the sample of earthquake survivors in Taiwan, bereaved women had three times the risk of developing a major depressive disorder among bereaved men [18]. Factors contributing to the gender differences in bereavement effects include living alone, normative circumstances of widows (women tend to live longer and are more likely to experience bereavement), physical health difference, social norms (women are more likely to be caregivers), and financial stress [26, 27]. Social support may also contribute to gender differences in the impact of bereavement on health [27].

### Impact of spousal bereavement on health outcomes in China

The existing literature includes a large number of studies on bereavement, mainly in industrialized settings including Western European countries, the US, and Japan [11, 28, 29]. Few studies have examined late-life spousal bereavement in developing countries, including China. Among the exceptions, Li and colleagues have surveyed a sample of older adults in Wuhan, China, during 1991–1994, and found that bereaved individuals were more likely to have worse mental health outcomes [16]. Assessing the impact of living arrangements on mental health among a sample of bereaved older adults in China, Ye finds that survivors who live independently in the same community with their children or grandchildren had better mental health conditions than those who live together with their children or grandchildren in the same household [30]. Jiao also estimated a statistically significant correlation between bereavement and mortality risk using a dataset from 1998 to 2005 in China [31].

However, the existing literature has not examined the impact of late-life spousal bereavement on health outcomes in China with a nationally representative data. There is also mixed evidence on the gender difference in the effect of spousal bereavement. While the gender difference has been observed in qualitative studies and an online survey in China [25, 32, 33], Li and colleagues report no evidence of a gender difference in the spousal bereavement effect using data from Wuhan during the mid-1990s [16]. To assess the impact of spousal bereavement on health outcomes in the text of a developing country and to examine the gender difference in such impacts will provide a critical update to the literature.

Therefore, this paper aims to examine the gender differential impact of spousal bereavement on health outcomes using updated nationally representative data from the China Health and Retirement Longitudinal Study (CHARLS) 2011–2015. Health outcomes examined are the Center for Epidemiologic Studies Depression Scale (CES-D) 10-item score and self-assessed health. We hypothesize that spousal bereavement may lead to depressive symptoms and worse self-assessed health and that such effects may differ between older Chinese men and women. Gender differences in the effect of spousal bereavement on health outcomes may exist because of gender roles expectations [25], social support [32], and gender difference in coping with bereavement [19, 33].

## Methods

### Data

This study used data from the CHARLS, a nationwide survey program aiming to provide quality data on the social, economic, health behavior, and health outcome-related information among the middle- and old-aged residents in China. The CHARLS survey covered community-dwelling residents from 450 villages and 150 counties in 28 provinces in China randomly chosen with a probability-proportional-to-size sampling technique from a sampling frame containing all county-level units except Tibet [34]. CHARLS intended to provide a high-quality public microdatabase with a wide range of information, including demographics, socioeconomic status, health condition, and healthcare utilization. To ensure quality and cross-study comparability of results, CHARLS was harmonized with leading international research studies, including the Health and Retirement Study [34].

The CHARLS had 3 years of primary surveys available to the public at the time of this research, i.e., 2011, 2013, and 2015. The respondents were residents aged 45 and over and their spouse (partner) regardless of age. The respondents entered the survey as a couple if the partner was alive or can be reached in 2011. The baseline survey in 2011 covers 17,596 residents, while the follow-up studies in 2013 and 2015 surveyed 18,455 and 20,967 residents, respectively. Increases in sample size included follow-ups with the non-responders in earlier years and refresh samples [35]. In addition, CHARLS conducted a life history survey in 2014 that provides information including childhood experience, health and healthcare history, and work history but not on current health conditions. Because of this limitation, we were unable to include the 2014 survey in our primary analysis. However, the demographic information in the 2014 survey can be used in constructing the bereavement indicator [36].

We focused on respondents who entered the survey with a partner so that the effect of spousal bereavement could be identified. We dropped the 3274 respondents who did not have a partner in 2011. We excluded 53 individuals who remarried within the 4 years of the study period because we were unable to ascertain if the remarrying was due to bereavement or divorce. We also excluded those with missing values of “spouse live/death status” and households whose reported spousal live/death status is inconsistent with their spouse mortality status recorded by the survey team, resulting in 11,035 households from which we identified the bereaved individuals and their matched control group.

### Variables

We defined the bereavement group as the respondents who experienced spousal bereavement during the CHARLS survey. That is, we needed to observe that the respondent’s spouse was alive in 2011 but passed away in 2013, 2014, or 2015. We identified 286 bereavements in 2013, 262 in 2014, and 225 in 2015, for a total of 773 bereavements.

CHARLS used a 10-item CES-D instrument to assess depressive symptoms by asking respondents about their positive feelings, negative emotions, and somatic symptoms during the last week. Responses to the questions are on a four-scale metrics coding from 0 to 3, and the total score ranges from 0 to 30, with higher scores indicating more depressive symptoms. CES-D 10 has been validated among older adults in China [37, 38].

We used self-reported health as an indicator of the quality of life. Quality of life summarizes the state of the remaining life free of impairment, disability, or handicap. It could also be interpreted as the degree to which persons perceive themselves able to function physically, emotionally, and socially [39]. Self-assessed health has been widely adopted in the measurement of the quality of life. The commonly used question to assess self-reported health was, “Is your health excellent, very good, good, fair, or poor?” The survey instrument is simple but has high reliability and validity [40].

Both the CES-D score and the self-assessed health were reported in 2011, 2013, and 2015, providing an opportunity to assess the impact of bereavement that occurred in 2013, 2014, and 2015. Because such impacts may have temporal patterns, including diminishing or strengthening over time, we included a set of year-after-bereavement variables. For simplification, we assumed that those who reported bereavement in 2013 lost their spouse in 2012. Therefore, the year after bereavement indicator can be 1 for those bereaved in 2013 and can be 1, 2, or 3 in 2015 because we could determine if bereavement occurred in 2014 using the CHARLS Life History Survey.

With longitudinal data, controlling the individual heterogeneities was essential to minimize the impact of reverse causality. However, we were unable to include the individual fixed effects, which will absorb the bereavement effect. Hence we controlled several key characteristics in the baseline year (2011), including being employed, being told having diabetes, and being told having heart disease in 2011. We included rural residence as an explanatory variable as previous studies have suggested differences in mental health prevalence and its correlates between rural and urban China [38]. Survivors’ education and social support received have been known to modify the effect of bereavement on health outcomes [32, 41]. We include a binary indicator of survivors’ education (1 if the survivor received at least 5 years of schooling, 0 otherwise) and an indicator if the survivor has received any public assistance (1 if yes, 0 otherwise).

To assess the gender difference in the impact of spousal bereavement, we included gender as an explanatory variable in the pooled sample. When a stratified analysis is justified, we used the gender-specific results.

### Analytic methods: propensity scoring matching

The empirical literature on bereavement has adopted the use of propensity score matching and difference-in-difference (DID) model. The DID model controlled for the unobserved common factors in the estimation of bereavement. Stephen and colleagues used propensity score matching, and a two-part DID model to estimate the socioeconomic cost of bereavement in Scotland [42]. They estimated the additional inpatient days of the bereaved survivor group, and then multiply it with the estimated daily inpatient cost to calculate the additional medical care burden.

We followed the literature to adopt a two-step procedure in estimating the health impact of bereavement. We first used propensity score matching to balance observed covariates between subjects from the treatment and control groups [43]. The control group should be similar to that of the bereaved group in important characteristics, such as the demographics and baseline health status. Controlling those confounding factors helped us to reduce the biases associated with the fact that the survivors may tend to be older and suffer from multiple chronic conditions.

Logistic regression was used to calculate the propensity scores. The logistic regression model estimated the probability that an individual experienced spousal bereavement. We followed a previous study [44] in selecting the candidate characteristics for estimating propensity scores, including age, gender, years of education, number of children, employment status, household size, region, and spousal demographics including spouse’s age, spouse’s education, and spouse’s working status in the baseline year (2011). Other than these factors, health conditions (ever had high blood pressure, ever had diabetes, and ever had heart problem) of both spouse and the respondent were included in obtaining propensity scores. We used stepwise regression for model selection to achieve a parsimonious specification. Details of the matching process are available from the authors upon request.

We identified a matched control for each respondent in the bereaved group by using the nearest neighbor method. This model is a standard tool that randomly selects N residents in the control group whose propensity score is within a certain “radius” of the propensity score for members of the bereaved group. Following the usual practice, we choose N to be 1 and set the radius to be 0.001. We pooled the bereaved and the matched control groups together for the primary analyses.

### Analytic methods: difference-in-differences

We used ordinary least squares for CES-D scores and ordinal logit for self-assessed health. We included an interaction between bereavement and time duration after bereavement in the regression equations to implement the DID regression models. Thus, our model was

$$ {\left( CES-D\right)}_{it}={\beta}_0+{\beta}_1\times Ber{e}_{it}+\left({\boldsymbol{AfterBere}}_{it}\times Ber{e}_i\right)\times {\boldsymbol{\beta}}_2+{\boldsymbol{Z}}_{\boldsymbol{it}}\times \boldsymbol{\varGamma} +{\varepsilon}_{it} $$

$$ o Logit{(SAH)}_{it}={\beta}_0+{\beta}_1\times Ber{e}_{it}+\left({\boldsymbol{AfterBere}}_{it}\times Ber{e}_i\right)\times {\boldsymbol{\beta}}_2+{\boldsymbol{Z}}_{\boldsymbol{it}}\times \boldsymbol{\varGamma} +{\varepsilon}_{it} $$

where CES-D is the 10-item CES-D score that ranges between 0 and 30 with higher scores indicating more depressive symptoms, and SAH is a five-category self-assessed health status with larger values indicating worse self-assessed health. *Bere* is the dummy variable indicating the respondent has experienced bereavement during the survey time period, *AfterBere* is a vector of the number of years after spousal death for individual *i* in year t. ***Z***_***it***_ is a vector of confounding variables and ***Γ*** its coefficient vector. All regressions were weighted using the sampling weights provided in the CHARLS database. Standard errors were robust errors clustered at the person level.

To determine if the gender difference exists, we included an interaction term between bereavement and the female gender. The analysis will be stratified by gender if we reject the null hypothesis that the coefficient of the interaction term is zero.

## Results

### Matched data

We identified 785 respondents who were bereaved before they were enrolled in the survey or bereaved during the study period (2011–2015). We dropped those respondents who were already bereaved when they entered the survey, which leaves 773 respondents remaining in the sample. We further limited the sample to those who provided information in all 3 years (2011, 2013, and 2015) so that we could examine the impact of bereavement (*n* = 682). Among those, the nearest neighbor algorithm matched 651 residents from the control group to the bereaved group. After excluding those with missing values in residential status (rural), we have 643 respondents in the bereaved group and 643 in the matched control group each year for 2011, 2013, and 2015.

We compared the key characteristics of the two groups to assess the effectiveness of the matching. Table 1 provides a comparison of the two groups in the two outcome variables and key explanatory variables. Results from the t-tests suggest that the two samples are similar in variables except for the mental health outcome (CES-D score) and receiving public assistance. Hence the matched control sample is similar to the bereaved sample in observed attributes.

**Table 1:** Comparing characteristics of the bereaved and matched control groups, China Health and Retirement Longitudinal Study 2011–2015

	Bereaved	Control	t-stat	*p*-value	Cohen’s D
CES-D (3 waves)	10.042 (6.951)	8.522 (6.281)	6.6	< 0.001	0.229
Self-Assessed Health (3 waves)	3.078 (0.943)	3.081 (0.947)	0.1	0.940	0.003
Woman	64.5%	64.2%	0.2	0.840	0.006
Working in Baseline Year	59.1%	58.0%	0.7	0.493	0.022
Rural Hukou	81.5%	83.7%	1.8	0.075	0.057
Having diabetes in baseline year	5.9%	7.2%	1.6	0.118	0.050
Having heart disease in baseline year	12.9%	11.0%	1.8	0.074	0.057
More than 5 years schooling	7.6%	8.9%	1.4	0.160	0.045
Receiving public pension/assistance	27.5%	24.4%	2.2	0.028	0.071
Observations	1929	1929			

(1) Standard deviation in parentheses; (2) CES-D: Center for Epidemiologic Studies Depression Scale; (3) t-statistics, p-value, and Cohen’s D are for comparing bereaved and matched control groups of the estimation sample

We stratified the sample into men and women to test for gender differences. Results in Table 2 confirmed gender differences in health outcomes and demographics. Women had higher CES-D scores than men, worse self-reported health, a lower percentage of employment, a higher percentage of being told to have diabetes in the baseline year (2011), and a lower proportion of having at least 5 years of schooling.

**Table 2:** Summary statistics: Men vs Women in the pooled bereaved and match control groups, the China Health and Retirement Longitudinal Study 2011–2015

	Total	Men	Women	*t*-stat	*p*-value	Cohen’s D
CES-D (3 waves)	9.285 (6.668)	7.654 (5.926)	10.180 (6.881)	10.7	< 0.001	0.385
Self-Assessed Health (3 waves)	3.080 (0.945)	2.966 (0.948)	3.142 (0.938)	5.3	< 0.001	0.187
Bereaved	50.0%	49.8%	50.1%	0.2	0.840	0.007
# years after spousal bereavement	0.855 (1.081)	0.823 (1.061)	0.873 (1.091)	1.4	0.169	0.046
Woman	64.4%					
Working in Baseline Year	58.6%	64.6%	55.2%	5.7	< 0.001	0.192
Rural Hukou	82.6%	78.2%	85.0%	5.4	< 0.001	0.181
Having diabetes in baseline year	6.5%	4.4%	7.7%	4.1	< 0.001	0.136
Having heart disease in baseline year	12.0%	12.2%	11.8%	0.4	0.720	0.012
More than 5 years schooling	8.2%	12.9%	5.7%	7.9	< 0.001	0.264
Receiving public pension/assistance	26.0%	27.0%	25.4%	1.1	0.278	0.036
Observations	3858	1374	2484			

(1) Samples are pooled from a bereaved group and its matched control group; (2) CES-D: Center for Epidemiologic Studies Depression Scale; (3) t-statistics, p-value, and Cohen’s D are for comparing men and women of the estimation sample

### Regression results

Results from the regression on CES-D scores in Table 3 suggested that the bereaved respondents have higher CES-D scores in the 2 years after their spouse passed away, with a peak at 2 years after bereavement, i.e., the coefficient for the interaction term between bereaved and year 2 after bereavement is the largest. Women had higher CES-D scores than men, indicating more depressive symptoms. Rural residential status was associated with about 1.940 points (about 0.290 times of the standard deviations of the CES-D score shown in Table 2) higher than those of urban residential status. Health status in the baseline year (2011), particularly having heart disease, was associated with higher CES-D scores; therefore, those having health issues in 2011 were more likely to be depressed. The interaction term between bereavement and woman is borderline statistically significant (*p*-value = 0.070), which, along with our interest in assessing the gender difference, justifies separate analyses by gender.

**Table 3:** Impact of bereavement on mental health, the China Health and Retirement Longitudinal Study 2011–2015

Variables	Coefficient	SE	*p*-value
Bereaved	0.440	(0.483)	0.363
Year 1 after bereavement among bereaved	1.292^**^	(0.657)	0.049
Year 2 after bereavement among bereaved	2.660^***^	(0.858)	0.002
Year 3 after bereavement among bereaved	0.267	(0.749)	0.722
Year 1 after Bereaved	0.030	(0.473)	0.949
Year 2 after Bereaved	−0.147	(0.561)	0.793
Year 3 after Bereaved	0.371	(0.563)	0.509
Woman	1.832^***^	(0.388)	< 0.001
Working in Baseline Year	−0.095	(0.268)	0.724
Rural Hukou	1.940^***^	(0.411)	< 0.001
Having diabetes in baseline year	1.461^**^	(0.585)	0.013
Having heart disease in baseline year	2.935^***^	(0.452)	< 0.001
Bereaved * Women	0.951^*^	(0.524)	0.070
More than 5 years schooling	−0.745	(0.543)	0.170
Receiving public pension/assistance	−0.402	(0.329)	0.221
Constant	5.233^***^	(0.469)	< 0.001
R-squared	0.095		

(1) Results are from weighted linear regressions; (2) Robust standard errors clustered at person level are in parentheses; (3) *** *p* < 0.01, ** *p* < 0.05, * *p* < 0.1; (4) CES-D: the Center for Epidemiologic Studies Depression Scale, 10 item

Tables 4 and 5 provided the regression results on CES-D scores for men and women, respectively. The coefficient of bereavement interaction terms on CES-D scores appeared to be significant only for 1 year after bereavement among men and marginally significant in the 3rd year after bereavement. In contrast, the effect among women was significant for the main bereavement effect (an increase of 1.520 points, or 0.228 standard deviations of the CES-D score) and an interaction effect occurred 2 years after bereavement, with the latter having the larger magnitude of impact (3.549 points or 0.532 standard deviations of the CES-D score). Having a rural Hukou (residence registration) was associated with higher CES-D scores among both men and women. Working in 2011 had protective effects among men but not women. Having diabetes in 2011 was associated with higher CES-D scores among men but not women. Having heart disease in 2011 was associated with higher CES-D scores among both men and women.

**Table 4:** Impact of bereavement on Mental health among men, the China Health and Retirement Longitudinal Study 2011–2015

Variables	Coefficient	SE	*p*-value
Bereaved	0.186	(0.545)	0.733
Year 1 after bereavement among bereaved	2.276^**^	(1.027)	0.027
Year 2 after bereavement among bereaved	1.113	(1.307)	0.395
Year 3 after bereavement among bereaved	2.083^*^	(1.239)	0.093
Year 1 after Bereaved	−0.310	(0.761)	0.684
Year 2 after Bereaved	0.091	(0.904)	0.920
Year 3 after Bereaved	−0.486	(0.946)	0.608
Working in Baseline Year	−0.628	(0.423)	0.138
Rural Hukou	1.766^***^	(0.503)	< 0.001
Having diabetes in baseline year	3.316^***^	(1.271)	0.009
Having heart disease in baseline year	2.310^***^	(0.746)	0.002
More than 5 years schooling	−0.755	(0.677)	0.265
Receiving public pension/assistance	−0.693	(0.583)	0.234
Constant	5.942^***^	(0.525)	< 0.001
R-squared	0.063		

(1) Results are from weighted linear regressions; (2) Robust standard errors clustered at person level are in parentheses; (3) *** *p* < 0.01, ** *p* < 0.05, * *p* < 0.1; (4) CES-D: the Center for Epidemiologic Studies Depression Scale, 10 item

**Table 5:** Impact of bereavement on Mental health among women, the China Health and Retirement Longitudinal Study 2011–2015

Variables	Coefficient	SE	*p*-value
Bereaved	1.520^***^	(0.426)	< 0.001
Year 1 after bereavement among bereaved	0.789	(0.835)	0.345
Year 2 after bereavement among bereaved	3.549^***^	(1.099)	0.001
Year 3 after bereavement among bereaved	−0.547	(0.925)	0.554
Year 1 after Bereaved	0.245	(0.591)	0.678
Year 2 after Bereaved	−0.298	(0.690)	0.666
Year 3 after Bereaved	0.852	(0.680)	0.211
Working in Baseline Year	0.072	(0.337)	0.830
Rural Hukou	2.158^***^	(0.581)	< 0.001
Having diabetes in baseline year	1.046	(0.660)	0.113
Having heart disease in baseline year	3.296^***^	(0.566)	< 0.001
More than 5 years schooling	−0.690	(0.845)	0.414
Receiving public pension/assistance	−0.250	(0.373)	0.502
Constant	6.654^***^	(0.585)	< 0.001
R-squared	0.070		

(1) Results are from weighted linear regressions; (2) Robust standard errors clustered at person level are in parentheses; (3) *** *p* < 0.01, ** *p* < 0.05, * *p* < 0.1; (4) CES-D: the Center for Epidemiologic Studies Depression Scale, 10 item

Table 6 presented the result of the ordinal logistics regression model of self-assessed health on bereavement and confounding variables. The pooled sample did not show a correlation of statistical significance between bereavement and self-assessed health. Employment in the baseline year and having at least 5 years of schooling had protective effects, but rural residential status and health problems in the baseline year were associated with a higher likelihood of reporting ill health. The interaction between bereavement and woman was not significant, and we did not stratify the analysis by gender.

**Table 6:** Impact of bereavement on self-assessed health, the China Health and Retirement Longitudinal Study 2011–2015

Variables	Coefficient	SE	*p*-value
Bereaved	0.093	(0.242)	0.703
Year 1 after bereavement among bereaved	−0.238	(0.212)	0.262
Year 2 after bereavement among bereaved	−0.070	(0.257)	0.786
Year 3 after bereavement among bereaved	0.293	(0.236)	0.216
Year 1 after Bereaved	0.238	(0.166)	0.153
Year 2 after Bereaved	−0.109	(0.173)	0.529
Year 3 after Bereaved	−0.199	(0.181)	0.272
Woman	0.295	(0.192)	0.124
Working in Baseline Year	−0.395^***^	(0.087)	< 0.001
Rural Hukou	0.475^***^	(0.121)	< 0.001
Having diabetes in baseline year	0.908^***^	(0.150)	< 0.001
Having heart disease in baseline year	1.031^***^	(0.114)	< 0.001
More than 5 years schooling	−0.343^**^	(0.160)	0.033
Receiving public pension/assistance	−0.063	(0.090)	0.486
Bereaved * Women	−0.048	(0.220)	0.829
/cut1	−2.114^***^	(0.316)	< 0.001
/cut2	−0.861^***^	(0.237)	< 0.001
/cut3	1.473^***^	(0.218)	< 0.001
/cut4	3.466^***^	(0.228)	< 0.001
Model fit: *F* = 13.76, Prob>F = < 0.001			

(1) Results are from weighted ordinal logit regressions

(2) Robust standard errors clustered at person level are in parentheses

(3) *** *p* < 0.01, ** *p* < 0.05, * *p* < 0.1

(4) Self-assessed health: 1.Very Good, 2.Good, 3.Fair, 4.Poor, 5.Very Poor

## Discussions

This study aims to assess the impacts of bereavement on mental health and self-assessed health among older adults who experienced spousal bereavement and the differences of such impacts between men and women. Specifically, we examine how mental health (as proxied by CES-D score) and self-reported health may change after bereavement among the bereaved. We have identified gender differences in the effect of bereavement on mental health but not on self-assessed health. Our results provide important implications on social support and policies for the bereaved older Chinese because the mental health sequelae of bereavement predict long-term physical health decline among older adults [45].

Our results indicate a stronger impact of bereavement on mental health among women than among men, and the timing of such impacts differs. While bereaved men experience worse mental health only in the first year after bereavement, bereaved women have much higher CES-D scores the second year after bereavement, in addition to a significant main bereavement effect. These results provided implications different from several previous studies, particularly those among high-income countries, where the impact of bereavement on health outcomes is more pronounced among men than among women [42, 46, 47]. Previous studies have observed a stronger effect of bereavement on mortality [42, 46] and more severe impact on emotion and happiness [47] among men. However, examining the impact of bereavement on health among Taiwanese older adults, one study finds that bereaved men have worse self-assessed health while bereaved women have higher CES-D scores [44]. Our results seem to be consistent with the Taiwan study because its target population shares the same cultural roots with mainland Chinese. The short-term impact in the first year after bereavement among men vs. the long-term impact of the main bereavement effect among women may be an indication that men could better adapt to the bereavement in the long term.

A host of factors may contribute to the gender difference in the impact of bereavement on health. Living arrangements, gender biological differences, gender role expectations, and social policies may have contributed to such differences. Longer life expectancies combined with the social norm in China of men marrying women of younger age have contributed to the fact that women may be more likely to experience bereavement— Table 1 has shown 64.5% of bereaved are women. Note that Table 2 shows a similar percentage of bereaved between men and women because sex was included in constructing the propensity score. The following factors may have contributed to a higher prevalence of depressive symptoms among bereaved women than men. First, women employed in formal sectors in China have a lower mandatory retirement age of 55 than men, who usually retire at age 60 [48]. Women may accrue lower pension over their careers than men and have less influence in the household budget [49]. Second, women are more likely to suffer income loss after bereavement because traditional social norms would encourage working men to marry stay-at-home women, but not vice versa. This argument is supported by the fact that 55.2% of women vs. 64.6% of men were working in 2011 (see Table 2). Our results have shown that women are more likely to have rural residential status – 85.0% of women vs. 78.2% of men had rural Hukou registration. Third, we suspect that social isolation, or living alone, may contribute to the gender difference in the bereavement effect as well because men tend to have a more extensive social network. Prior studies have shown the importance of social participation for mental health among older adults in a variety of settings [21, 38, 50]. Chinese women tend to have a much smaller social network, which results in much fewer resources available for social support than their men peers [51]. Fourth, social norms on gender roles may have contributed to the gender difference in the incidence of depressive symptoms by introducing different expectations after bereavement [16, 25, 26, 52]. Consistent with those gender disparities, our results indicate that older Chinese women are more likely to suffer from depression after bereavement, and the impact may last for 2 years after bereavement. Fifth, another underlying reason may be attributed to different coping behaviors between men and women [11, 19, 33]. Compared with their men peers who have a task-oriented coping strategy, women are more likely to take emotion-focused copying styles [53], which may result in women experience more psychiatric symptoms.

The effects of rural residence on mental health and self-reported health deserve special attention. China has undergone an economic transition accompanied by a dramatic urbanization, leading to able-bodied young adults moving to urban areas for economic opportunities and leaving older adults and sometimes children behind in rural areas. Such population mobility has important implications for rural social support and the healthcare system [54–56]. Older adult survivors residing in rural areas are more likely to have depressive symptoms because of a lack of social support, poor economic security, and social isolation [16, 38]. They may report poor health due to prevalent chronic conditions and inadequate access to healthcare [57].

Beyond the empirical findings, this study may have important policy implications for China’s older adult care and pension systems. China does not have a formal survivor benefits program for those in the informal sector or the unemployed; thus, bereaved older women often rely on savings or children for support. China’s New Rural Pension Program has protected the enrolled but also crowded out support from adult children [56]. Recent research also points to the inadequate enrollment of the program [58]. Well-designed survivor benefits may offer protection against income shocks and help in terms of the funeral arrangement of the deceased that could reduce the stress among the bereaved, particularly rural women.

To our knowledge, this paper is the first research to assess the impact of bereavement on health outcomes among a sample of mid- to old-age Chinese adults. We used propensity scoring matching to reduce the bias associated with the correlation between the health status of the deceased and the bereaved. However, this paper does have four important limitations. First, we choose not to assess the mortality effects of bereavement because the CHARLS has a relatively short time span (5 years) and a moderate sample size of the bereaved; as a consequence, death after bereavement is a rare event in this sample. We did not examine the medical expenditure associated with bereavement, which merits a separate in-depth analysis because it involves income loss, support from children, and transfer income via pension and survivor benefits that have significant variation across regions. Second, we did not model the potential lag between bereavement and health outcomes because the two outcomes, mental health and self-assessed health, are more responsive to external events than mortality and other chronic conditions. The uneven interval and a limited number of years of the CHARLS survey also made it challenging to model lagged impacts. With future waves of the CHARLS, we may be able to model the lagged impact of bereavement on mortality and other chronic outcomes, including cancer and diabetes. Third, we are unable to ascertain the exact time when the bereavement occurred; thus, the time after bereavement suffers from measurement errors. Unfortunately, we are unable to address this data limitation in the secondary data analysis. Fourth, we did not model survivor benefits and pension income explicitly as regional policies vary. We intend to explore the availability of geographic identifies and further examine the impact of such policies when data are available.

## Conclusions

This paper finds that bereavement affects men and women differently, with a stronger impact on depressive symptoms among bereaved women. Bereavement is not associated with self-reported health. Rural residence (Hukou registration), and health condition, proxied by having heart disease in baseline year, are important correlates of depressive symptoms and self-reported health.

Our results support further research on survivor benefits for the disadvantaged populations in China, including women, rural residents, and those who are unemployed or employed in informal sectors. Survivor benefits programs may protect them from harmful impacts of the loss of a family member on their mental health outcomes and quality of life in the short-run and potentially mortality and chronic conditions in the long-term.

## Data Availability

Data will be available from the corresponding author (ZC) upon request after publication.

## Abbreviations

CHARLS: The China Health and Retirement Longitudinal Study
CES-D: Center for Epidemiologic Studies Depression Scale
DID: Difference-in-difference
